# Clogging and Water Quality Change Effects of Typical Metal Pollutants under Intermittent Managed Aquifer Recharge Using Urban Stormwater

**DOI:** 10.3390/ijerph182413272

**Published:** 2021-12-16

**Authors:** Siyao Ma, Yalin Song, Xueyan Ye, Xinqiang Du, Jingjia Ma

**Affiliations:** 1 Key Laboratory of Groundwater Resources and Environment, Ministry of Education, Jilin University, Changchun 130021, China; macy20@mails.jlu.edu.cn (S.M.); DG20290044@smail.nju.edu.cn (Y.S.); duxq@jlu.edu.cn (X.D.); 2 College of New Energy and Environment, Jilin University, Changchun 130021, China; 3 School of Earth Science and Engineering, Nanjing University, Nanjing 210023, China; 4 Shenyang Engineering Company, China Coal Technology & Engineering Group, Shenyang 110015, China; majingjia83@gmail.com

**Keywords:** clogging, water quality change, urban stormwater, intermittent MAR, metal contamination

## Abstract

Managed aquifer recharge (MAR) using urban stormwater facilitates relieving water supply pressure, restoring the ecological environment, and developing sustainable water resources. However, compared to conventional water sources, such as river water and lake water, MAR using urban stormwater is a typically intermittent recharge mode. In order to study the clogging and water quality change effects of Fe, Zn, and Pb, the typical mental pollutants in urban stormwater, a series of intermittent MAR column experiments were performed. The results show that the type of pollutant, the particle size of the medium and the intermittent recharge mode have significant impacts on the pollutant retention and release, which has led to different clogging and water quality change effects. The metals that are easily retained in porous media have greater potential for clogging and less potential for groundwater pollution. The fine medium easily becomes clogged, but it is beneficial in preventing groundwater contamination. There is a higher risk of groundwater contamination for a shallow buried aquifer under intermittent MAR than continuous MAR, mainly because of the de-clogging effect of porous media during the intermittent period.

## 1. Introduction

Managed aquifer recharge (MAR) is part of the palette of solutions to water shortage, water security, water quality decline, falling water tables, and endangered groundwater-dependent ecosystem [1]. The current global water shortage and continuously increasing demand mean that managed aquifer recharge (MAR) with high-quality surface water (rivers, lakes, reservoirs, etc.) cannot be carried out in many areas. However, MAR using urban stormwater shows great potential and broad prospects [2,3]. Compared to other water, e.g., municipal and industrial wastewater, that could be reclaimed, urban stormwater, as a MAR water source, has particular advantages, namely renewability and lower pollution degree [4,5], which make its usage more economical and sustainable. Many countries, such as Australia, the United States, and India, have successfully adopted stormwater as the recharge water source [6,7].

Common problems in MAR, using urban stormwater, include clogging of the filter media and water quality deterioration [8]. Clogging occurs when the permeability of the filter media decreases due to physical, chemical, and biological processes during recharge. The quality of the recharge water source may change during the infiltration process, affecting groundwater quality [9,10]. Clogging directly affects the efficiency, operational cost, and service life of the MAR facilities, whereas water quality change poses a risk to the groundwater environment and human health [11,12,13,14].

Numerous efforts have been made to solve the problems of filter media clogging and water quality change, respectively. However, only a few considered the interaction between clogging and water quality change. Maliva (2015) [15] pointed out that the performance of MAR systems is highly dependent upon local hydrogeology, which controls the movement and mixing of stored water and fluid-rock interaction, which can impact recharged water quality. Du et al. [16] conducted a laboratory MAR experiment using tap water and analyzed the relationship between media clogging, dissolution, and water quality change, where the clogging by metals such as iron and aluminum resulted in changes in the concentration of the corresponding substances in the infiltration water. Ye et al. [17] conducted MAR experiments to study the influence of the ionic strength on the clogging by suspended solids. Cui et al. [18] found that water quality improved via iron clogging of aquifer media. Zhang et al. [19] showed than 80% of the injected Fe (III) remained in the sand column, and more than 50% was retained within the upper 1 cm of the column inlet during MAR using stormwater, which indicated that the discharge water quality was affected by the clogging process. Due to a lack of systematic research, the interaction laws and its mechanisms between filter media clogging and water quality changes still hard to quantify clearly in the MAR process.

The quality of stormwater flowing through different underlying surfaces, e.g., roof, road, and natural land, is very different [4]. Stormwater pollutants mainly include nutrients, organic matter, suspended solids, and heavy metals, among which the most common include Zn, Pb, Cu, Cd, Fe, and Mn [4,20].

Due to the variation in the amount and spatiotemporal distribution of atmospheric precipitation, urban stormwater MAR is a typically intermittent recharge mode, which is different from the continuous recharge mode in the evolution law and clogging mechanism. Under intermittent MAR, saturated/unsaturated conditions and environmental parameters, such as the water content and redox potential in the filter media, change significantly, a fact on which we relied in this study. The interruption of recharge processes may further impact the transport and transformation of pollutants [21,22]. Therefore, Fe, Zn, and Pb, the typical metal pollutants in stormwater [4], were taken as examples to examine the interaction between the clogging of porous media and infiltration water quality changes in intermittent MAR using stormwater.

## 2. Materials and Methods

A series of Darcy flow experiments were conducted under intermittent flow and variably saturated conditions to study porous media clogging and water quality change by using urban water containing Fe, Zn, and Pb.

### 2.1. Filter Media and Recharge Water

The experimental filter media was river sand, in which the particle size of fine sand was 0.10–0.25 mm and that of medium sand was 0.25–0.5 mm. The mineral composition of the sand was tested using X-ray diffraction, and the results are shown in Table 1. In the process of preparation, river sand was soaked in 20% nitric acid solution for 24 h to remove metals, and it was washed with Milli-Q water later, until the pH was neutral.

Tap water mixed with analytical pure ferric chloride (FeCl_3_), zinc chloride (ZnCl_2_), or lead chloride (PbCl_2_) was used to prepare the recharge water source. The major quality characteristics were shown in Table 2. The concentration of the target pollutants was set based on the Quality Criteria for Groundwater Recharge of Urban Reclaimed Water Reuse (GB-19772-2005). The recommended concentrations of Fe and Zn were 3 and 1 mg/L, respectively. Considering the test accuracy, the concentration of Pb in the recharge water was set at 2 mg/L, which is 40 times the recommended value.

### 2.2. Experimental Setups and Experimental Schemes

**Figure 1:**
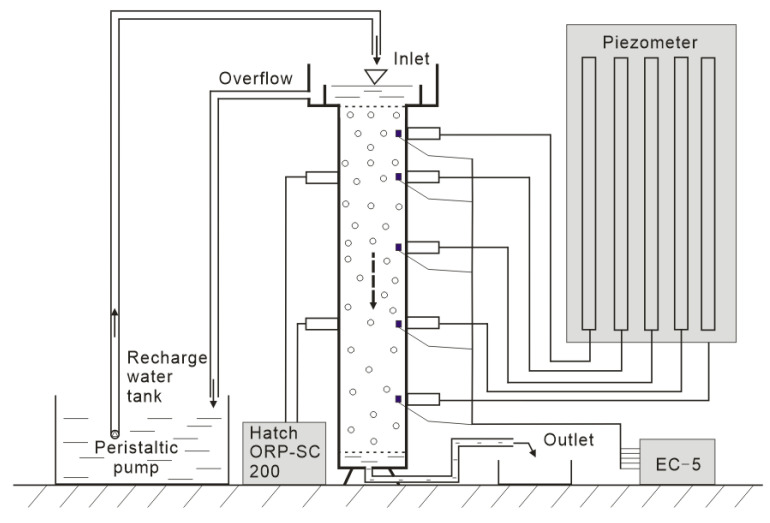
Physical diagram of the intermittent recharge experimental setups.

The experimental setup is shown in Figure 1, composed of a water supply tank, an infiltration column, a piezometer board, and a sampling system. The column was made of Perspex, had a length of 50 cm, and had a radius of 6 cm. The sand was installed by the wet packing method. The water levels of the sand column inlet and outlet were kept constant during the recharge process. The sand column was connected with the piezometer to observe the level of the simulated water table. The water content was measured continuously at 5, 10, 20, 30, and 40 cm away from the sand column inlet by an EC-5 sensor, which depicts the water content of the sand column by measuring the changes in the output voltage [23]. The redox potential of the water in the sand column was measured 10 and 30 cm away from the sand column inlet by the Hatch ORP-SC200. The discharge from the outlet was measured using a measuring cylinder.

The whole experiment process consisted of three recharge stages separated by two intermittences. The water was pumped from the tank into the water supply controller of the sand column, and the water above the specified level flowed back to the recharge water tank through the overflow port. The recharge water flowed through the sand column from top to bottom and flowed out from the specified elevation downstream outlet, which was set above the bottom of the sand column to maintain a threshold saturated zone during recharge intermittence. All the columns were flushed with about 3 pore volumes of background tap water before experiment. The first recharge process was started and continued until the outlet pollutant concentration reached a nearly constant level, and then, the inlet was turned off to start an intermittence for 3 days, which is a long enough duration for the environment conditions in the sand column to reach a new equilibrium [24]. After the intermittence, the recharge process was restarted and continued until another relatively stable outlet concentration of pollutants was reached. The second 3-day intermittence and the third recharge process were performed in the same way as the previous steps. Six groups of experiments were designed according to the purpose of the experiment (Table 3).

### 2.3. Hydraulic Conductivity

Darcy’s law was used to calculate the hydraulic conductivity of the sand column based on the data acquired from different recharge stages:

K = (QL)/(A × ∆h)(1)

where Q is the outlet flow rate (cm^3^/s), L is the distance between any two pressure monitoring points on the sand column (cm), A is the section area of the sand column (cm^2^), and ∆h is the head difference between any two piezometric points (cm).

The hydraulic conductivity (K) usually varies with time and space during managed aquifer recharge. K_0_ was defined as the initial hydraulic conductivity and the normalized hydraulic conductivity (K/K_0_) was calculated to represent the change in the permeability of the filter media. K/K_0_, in this research, also can be adopted to represent the flow rate variation during MAR experiment under the condition of the constant water level which hydraulic gradient (∆h/L) is fixed to be 1. K/K_0_ < 1 indicates that the sand column is clogged. The smaller the value of K/K_0_, the greater the sand column clogging degree.

### 2.4. Transport of Pollutants in the Sand Column

Water samples from the outlet were taken manually according to the recharge time and the degree of clogging. Sampling was more intensive at each initial stage of recharge. The concentration of Fe, Zn, or Pb in the water samples was detected using an atomic adsorption spectrometer after sampling. The normalized concentration (C/C_0_) was calculated by comparing the target pollutant concentration in the outflow (C) with that in the inflow (C_0_).

The retention mass of the pollutants in the sand column was determined at the end of the experiments. The sand column was dismantled by stratification after the experiment and took sand samples at 0–5, 5–10, 10–20, 20–30, 30–40, and 40–50 cm along the sand column. After drying and weighing the sand samples, they were soaked in centrifuge tubes that contained Milli-Q water. The tubes were shaken vigorously with a vortex mixer for 3 min to separate the retained materials, and then, we measured the concentration of metal ions in the solution and calculated the mass of the pollutants in each layer of the sand column.

## 3. Results and Discussion

The recharge process is a typical Darcy experiment in which the water discharge, water level, concentration of pollutants and environmental parameters such as redox and water content are all monitored. C/C_0_ and K/K_0_ can be derived based on the monitored data. At the end of the experiment, the metal retained in the sand column was measured and calculated to verify the experimental results.

### 3.1. Flow and Transport of Typical Metal Pollutants under Intermittent MAR

**Figure 2:**
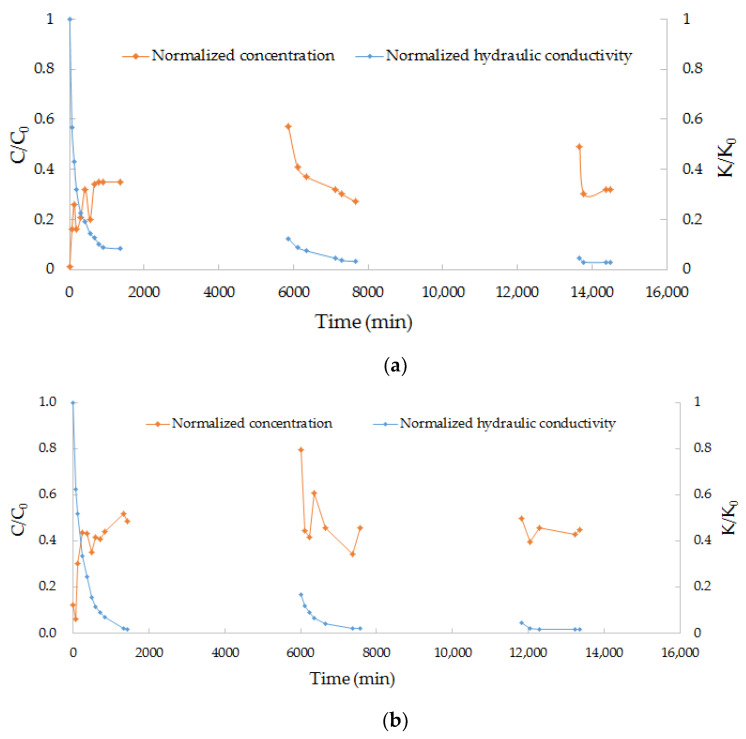

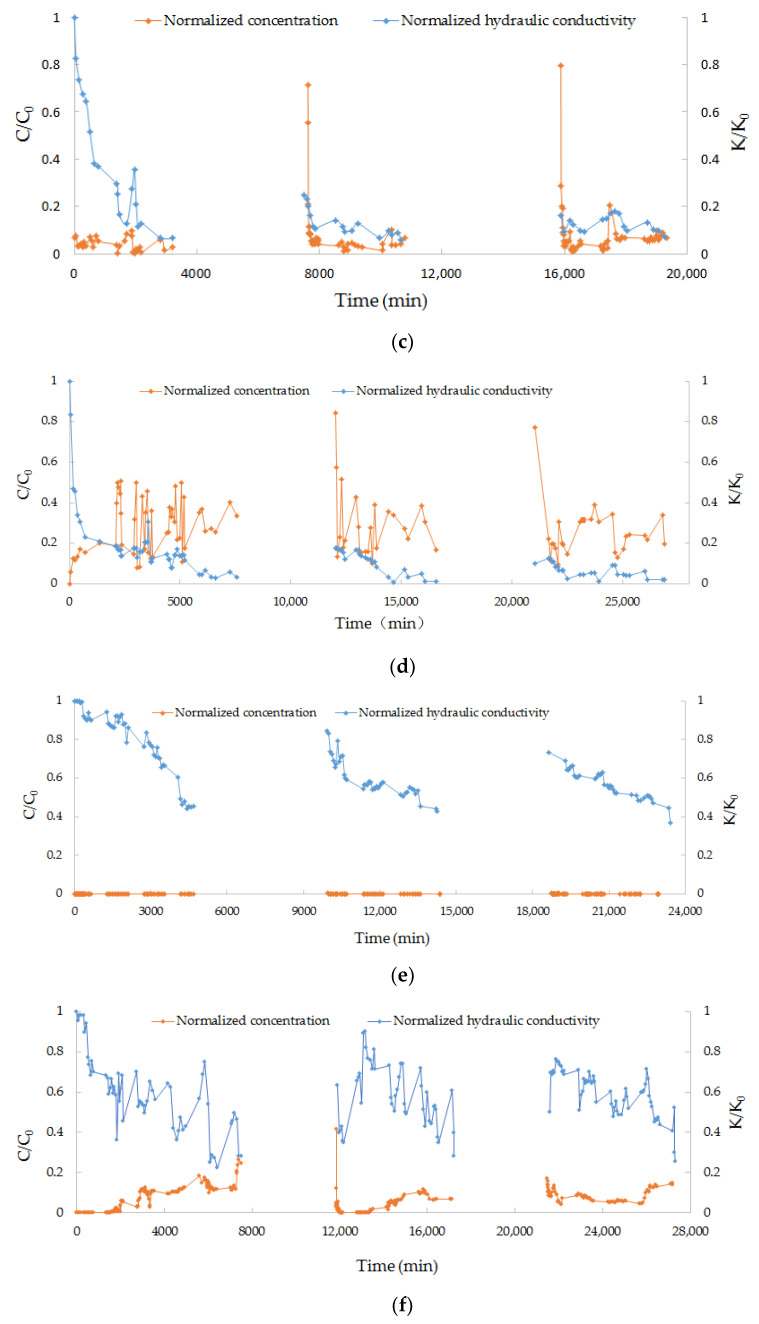
Diagram of K/K0 vs. C/C0. (**a**) E1 (Fe + fine sand); (**b**) E2 (Fe + medium sand); (**c**) E3 (Zn + fine sand); (**d**) E4 (Zn + medium sand); (**e**) E5 (Pb + fine sand); (**f**) E6 (Pb + medium sand).

The flow and transport laws of the pollutants during intermittent MAR are similar, as indicated by their discharge rate, which was represented by normalized hydraulic conductive (K/K_0_) and normalized concentration curves (C/C_0_) in all six experiments (Figure 2). In the first stage, the discharge decreased, and the C/C_0_ of the pollutants increased and reached a relatively stable value. At the beginning of the second and the third recharge process, q and C/C_0_ rose to 1.5–2.5 times higher value than the end of the previous stage, and then, it decreased very fast and approached the relatively stable value of the previous recharge stage. The discharge rate and normalized concentration (C/C_0_) of each pollutant in the coarser medium is generally higher than that in the fine medium.

For experiment E5 and E6, the discharge rate is higher than the other experiment with the same filter medium, whereas the normalized concentration is much lower than the other experiments. The results indicate that Pb is much easier to absorb on sand than Fe and Zn. Many literatures also reported the high removal efficiency of Pb in sand columns [24,25,26,27].

### 3.2. Retention of Typical Metal Pollutants under Intermittent MAR

**Figure 3:**
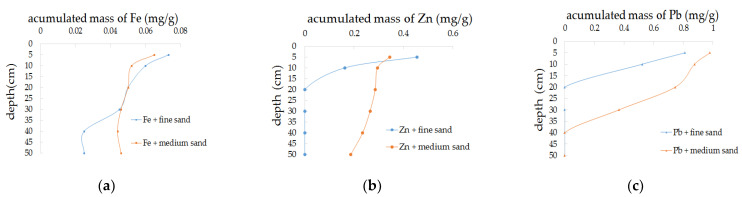
Cumulative mass distribution of pollutants in media with different particle sizes. (**a**) E1 and E2 (Fe); (**b**) E3 + E4 (Zn); (**c**) E5 + E6 (Pb).

By comparing the pollutants’ retention profiles in fine sand (Figure 3), we observe that the accumulated amount of the three pollutants in the fine sand was concentrated in the surface of the filter media. Table 4 presents experimental mass balance information, which is calculated from measurements of recharge and discharge. The rough estimation of mass balance showed a basic reliable range of measurement error. Apart from Fe in the sand column, the cumulative distribution of the other two pollutants in the fine sand, from 10 cm along the sand column to the bottom, was nearly zero, which indicates that the type of the pollutant has an impact on its accumulation in the sand column, and the migration ability of Fe was stronger than that of Zn and Pb. Eh and pH in the sand column were conducive to the formation of Fe(OH)_3_(s), which adsorbed on the filter media, meaning that retention was easier. Some of small-sized Fe(OH)_3_(s) entered the sand column with the water flow and were adsorbed on the medium, so the retention amount of Fe was large on the surface of the sand column and relatively uniform in its interior. This speculation is consistent with relevant research [28,29,30,31].

The potential of water–rock interactions between the recharge water and the filter media was evaluated. According to Zn and Pb content in the experiment, smithsonite (ZnCO3), cerussite (PbCO3), and anglesite (PbSO4) may be generated. The saturation index (SI) can be used to judge the dissolution and precipitation of minerals [32]. All the calculated SIs of possible minerals are less than zero, indicating that no minerals are generated in the process of recharge. Therefore, the accumulation of pollutants is mainly due to adsorption or interception.

### 3.3. Interaction of Clogging and Water Quality Change under Intermittent MAR

**Figure 4:**
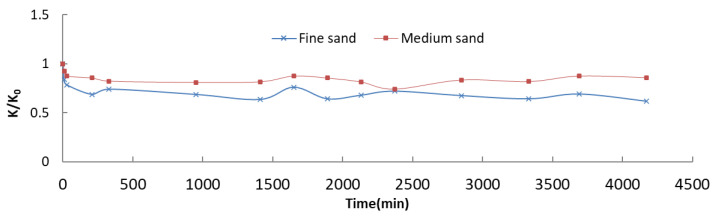
Clogging effect of background water.

In order to examine clogging effect of the pollutants, a blind experiment using tap water was conducted for reference (Figure 4). We compared the change in the hydraulic conductivity of the sand column and normalized the concentration of pollutants during the experiments; the results are shown in Figure 2 and Table 5.

Based on the blind experiment (Figure 4), the clogging can be caused by recharge using tap water. However, the contribution to clogging is no more than 40% and 20% within 4500 min for fine and medium sand, respectively. Figure 2 and Table 5 showed that the increase and decrease processes of pollutant normalized concentration (C/C_0_) were consistent with the change in the normalized hydraulic conductivity (K/K_0_) of filter media. In the first stage, with the inflow of recharge water, some metal might adsorb on the surface of the sand medium, meaning that the effective porosity of the filter media decreased more severe than only tap water circumstance, while the hydraulic conductivity decreased much faster than the blind experiment and became relatively stable. Clogging rapidly occurred inside the sand column, and the pollutant concentration in the outlet sample gradually stabilized. It should be noted that, in the early stages of the last two recharges, centralized release of the pollutants occurred. The main reason for this is that, during the intermittence, under the function of gravity and evaporation, the upper part of the sand column gradually lost water and changed from saturated to unsaturated, resulting in the potential destruction of the dense soil structure; namely, cracks may occur on the surface of the sand column, creating shortcut flow paths for the subsequent recharge water with a high concentration of pollutants. As recharge continued, the permeability of the medium gradually decreased, and the shortcut flow paths also vanished rapidly, meaning that the normalized concentration dropped quickly, gradually approaching a new relatively stable state.

### 3.4. Changes of Environmental Factors in the Sand Column

The sand column was saturated in the first recharge stage, but the upper part was switched to unsaturated during the intermittence. The changes were mainly embodied in the water content and redox potential.

### 3.5. Water Content

**Figure 5:**
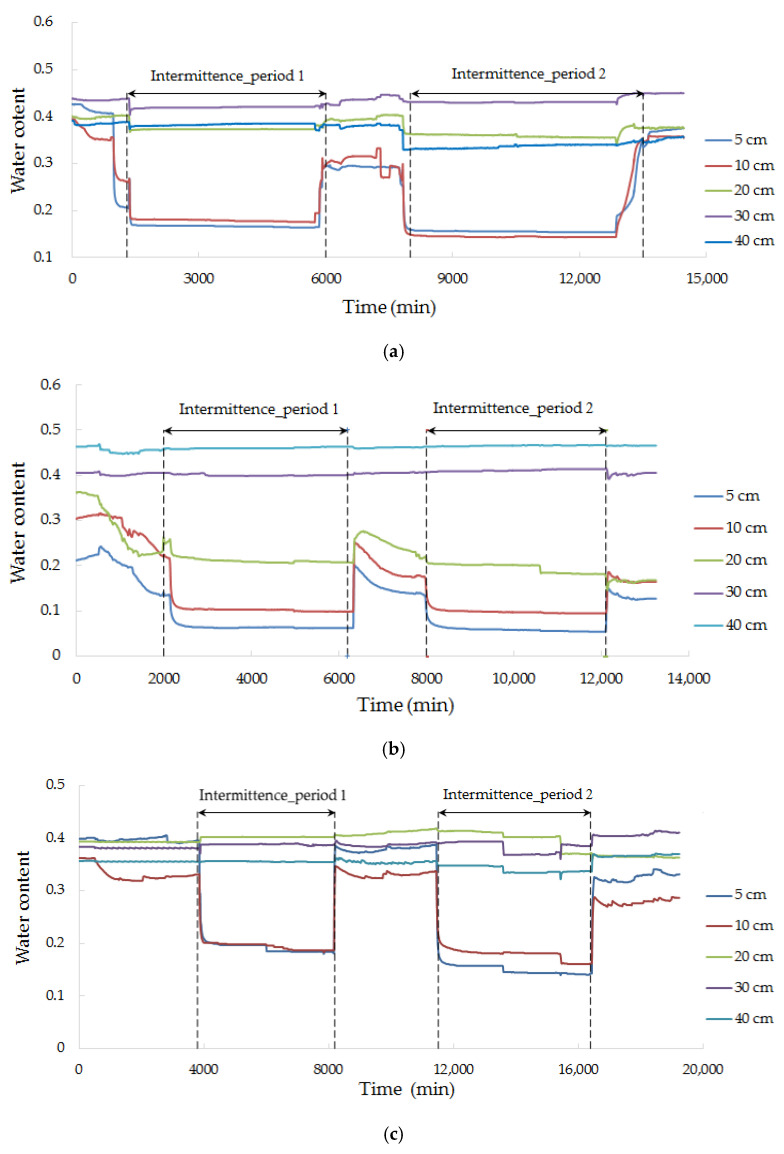

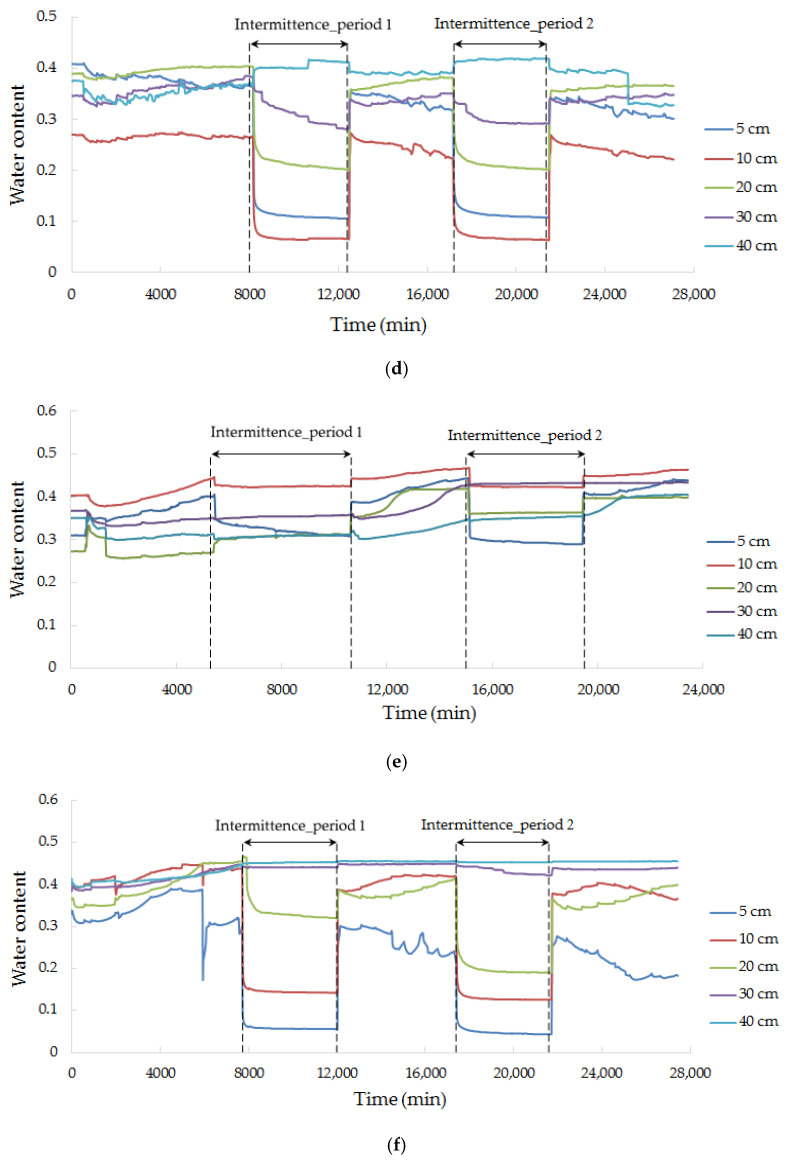
Water content change with time. (**a**) E1 (Fe + fine sand); (**b**) E2 (Fe + medium sand); (**c**) E3 (Zn + fine sand); (**d**) E4 (Zn + medium sand); (**e**) E5 (Pb + fine sand); (**f**) E6 (Pb + medium sand).

The sand columns were saturated before the experiment begin, and the volumetric water content was around 0.4 (Figure 5). The volumetric water content, at different positions of the sand column, was slightly different because the packing sand density was difficult to maintain consistent in such a relatively large sand column. The downward recharge flow process, which may induce air trapped into the sand column, might be another reason for the difference of water content.

In both medium and fine sand, during the first recharge, the water content kept steady because the sand column was water-saturated. After the first recharge, the water in the upper part of the sand column flowed downward, resulting in a change from saturated to unsaturated at the range of 20 cm from the inlet. The change of water content was little at 30 cm and 40 cm because the constant outlet head was kept at 5 cm above the bottom of the sand column, with a threshold of water and supporting capillary water (Figure 5). When the restarted recharging after the intermittence, the water content of the upper part of the sand column rose somewhat, but sometimes, it could not revert to the initial water content because air entered the medium during the intermittence, and it remained unsaturated.

### 3.6. Redox Potential

**Figure 6:**
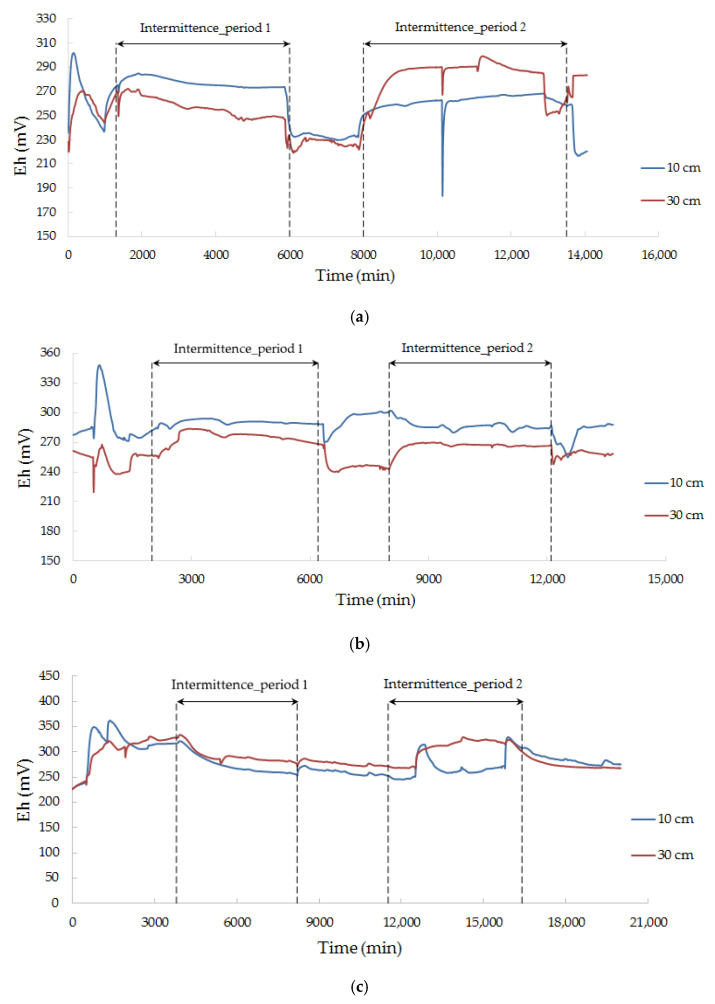

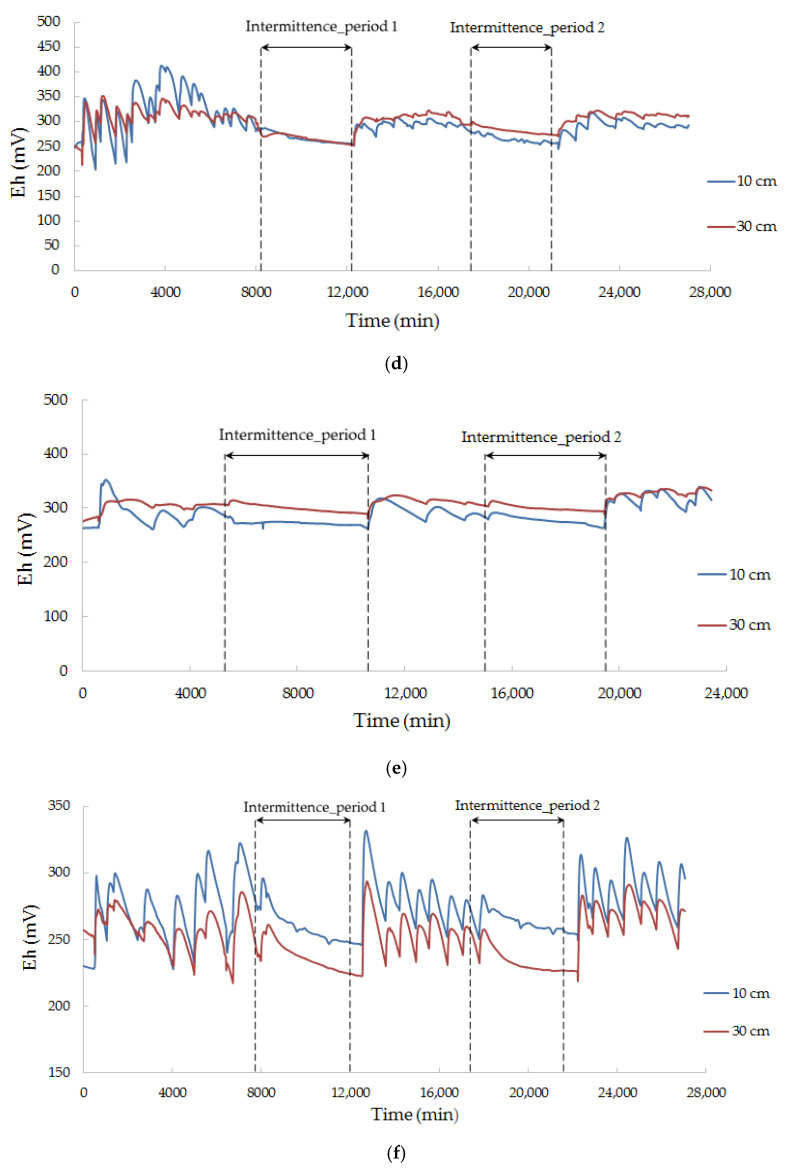
Redox potential change with time. (**a**) E1 (Fe + fine sand); (**b**) E2 (Fe + medium sand); (**c**) E3 (Zn + fine sand); (**d**) E4 (Zn + medium sand); (**e**) E5 (Pb + fine sand); (**f**) E6 (Pb + medium sand).

The redox potential of the recharge water is affected by the pollutant type in the water source (Figure 6). When the main pollutant was Fe, the redox potential of the medium gradually decreased as recharge began, possibly because Fe^2+^ was combined with oxygen to precipitate as iron hydroxide [33], and thus, the dissolved oxygen content of the recharge water was reduced, and the redox potential of the filter media was affected [34]. When the pollutant was Zn or Pb, the redox potential showed an upward trend during the recharge process, mainly due to the continuous contact between the recharge water and air under the magnetic stirrer, and the oxygen content of the water source increased slightly. The oxygen dissolved in the water decreased gradually during the intermittence, and the air entering the sand column supplemented the oxygen content. For the sand column, where surface clogging was less serious, there was more air entering the sand column. It has a greater impact on the redox potential of the medium than the oxygen dissolved in the water; thus, the redox potential presents an upward trend (Figure 6a,b). For the sand column, where serious surface clogging is greater, the rise of the redox potential, caused by air entering the sand column, was limited, and the dissolved oxygen content of the water dominated, so the redox potential decreased during the pause (Figure 6c–f).

## 4. Conclusions

Compared with continuous MAR, we mainly explore the clogging and water quality change effects of typical metal pollutants, under intermittent MAR, using urban stormwater. The type of porous media, the type of pollutants and the mode of recharge all impacted the water quality and media clogging. The conditions conducive to clogging are also those conditions conducive to the avoidance of groundwater pollution in MAR, such as porous media with finer particles and metals that are more likely to be retained in the media. The most important difference between intermittent and continuous MAR is that, in the former, the normalized concentration of pollutants reaches a higher peak value at the beginning of the recharge process after the intermittence, and the de-clogging effect of porous media was inferred as the main reason for this. The results indicate that there is a higher contamination risk of shallow groundwater under intermittent MAR. However, this conclusion is obtained under the specific laboratory conditions, and its rationality and reliability under real-world conditions remains to be verified, especially for the MAR with deep buried groundwater.

In this study, the reason for the increase in permeability at the beginning of the second and third recharge periods is derived from the experimental phenomena, which still need to undergo more detailed research to support our analysis. In addition, the following limitations in this research are as follows: (i) Soil CEC, clay content, organic matter content, flow rate, and many other factors that influence heavy metal retention in filter medium should be considered for a comprehensive study; (ii) a series of parallel experiments are needed to compare the results; (iii) there is still a gap between the experimental conditions including recharge water source and the real MAR scenarios.

## Figures and Tables

**Table 1:** Mineral composition of the filter media.

Mineral Name	Chemical Formula	Content (%)
Quartz	SiO_2_	21
Potash (alkaline) feldspar	KAlSi_3_O_8_	22
Plagioclase	Na[AlSi_3_O_8_]-Ca[Al_2_Si_2_O_8_]	52
Biotite	K(Mg,Fe)_3_AlSi_3_O_10_(F,OH)_2_	5

**Table 2:** Quality characteristics of the tap water.

Component	HCO_3_^−^	CO_3_^2−^	Cl^−^	SO_4_^2−^	NO_3_^−^	K^+^	Na^+^	Ca^2+^	Mg^2+^
Concentration(mg/L)	75.4	0.0	22.9	26.9	10.0	5.5	9.1	13.8	5.3

**Table 3:** Pollutant migration design under different conditions.

Group	Pollutant	Concentration (mg/L)	Medium	Hydraulic Gradient	Intermittent Time (d)
E1	Fe	3	Fine sand	1	3
E2	Fe	3	Medium sand	1	3
E3	Zn	1	Fine sand	1	3
E4	Zn	1	Medium sand	1	3
E5	Pb	2	Fine sand	1	3
E6	Pb	2	Medium sand	1	3

**Table 4:** Mass balance information from the column experiment. Here, M_In_ denotes the injection mass of pollutant; M_BTC_ and M_Ret_ denote the mass of the injection pollutant that was recovered in the breakthrough curve and retained in the sand column, respectively.

No.	M_In_ (mg)			M_BTC_ (mg)			M_Ret_ (mg)
	Recharge Stage			Recharge Stage			
	1st	2nd	3rd	1st	2nd	3rd	
E1	165.0	60.0	40.5	2.5	3.3	0.8	251.8
E2	184.5	105.0	60.0	9.2	7.0	2.2	325.8
E3	160.0	136.0	137.0	0.7	0.6	0.5	430.7
E4	815.0	380.0	425.0	13.7	7.1	9.7	1588.1
E5	296.0	267.0	269.0	66.2	28.7	60.6	675.7
E6	996.0	872.0	710.0	27.1	8.4	24.2	2509.1

**Table 5:** Corresponding characteristic values of C/C_0_ and K/K_0_.

Recharge Stage		E1		E2		E3		E4		E5		E6	
		Start	End	Start	End	Start	End	Start	End	Start	End	Start	End
1st recharge	C/C_0_	0	0.35	0	0.48	0	0.03	0	0.34	0	0.0008	0	0.26
	K/K_0_	1	0.08	1	0.02	1	0.06	1	0.03	1	0.45	1	0.28
2nd recharge	C/C_0_	0.57	0.30	0.80	0.46	0.71	0.04	0.58	0.17	0.0019	0.0003	0.41	0.07
	K/K_0_	0.09	0.03	0.17	0.02	0.25	0.07	0.18	0.01	0.79	0.43	0.63	0.28
3rd recharge	C/C_0_	0.49	0.32	0.50	0.45	0.08	0.03	0.77	0.20	0.0013	0.0007	0.17	0.14
	K/K_0_	0.05	0.03	0.04	0.02	0.16	0.07	0.10	0.02	0.73	0.37	0.50	0.26

## Data Availability

Data is contained within the article.
